# Profiling of victimization, perpetration, and participation: A latent class analysis among people with severe mental illness

**DOI:** 10.1371/journal.pone.0208457

**Published:** 2018-11-30

**Authors:** Wendy M. M. Albers, Diana P. K. Roeg, Yolanda Nijssen, Jaap van Weeghel, Inge M. B. Bongers

**Affiliations:** 1 Tranzo Scientific Center for Care and Welfare, Department of Social and Behavioral Sciences, Tilburg University, LE Tilburg, The Netherlands; 2 GGzE Center for Mental Health Care, AX Eindhoven, The Netherlands; 3 Parnassia Psychiatric Institute, Monsterseweg, RJ Den Haag, The Netherlands; 4 Phrenos Center of Expertise, BE Utrecht, The Netherlands; 5 Erasmus Center for Health Care Governance, Erasmus University, DR Rotterdam, The Netherlands; University of Sao Paulo Medical School, BRAZIL

## Abstract

**Background:**

Persons with severe mental illness are more prone to victimization and experience more difficulties regarding societal participation than other community members. These experiences vary greatly among individuals. Community mental health care should offer more individualized support by addressing these differences in experience. Therefore, this study aimed to identify subgroups of outpatients with severe mental illness based on their experiences of social participation and victimization.

**Methods:**

Data from patients with severe mental illness from eight outpatient teams in the Netherlands were used to perform latent class analysis. From the total caseload, 395 patients agreed to participate. Classes were based on: i) criminal victimization incidents, ii) criminal perpetration incidents (Dutch Safety Monitor), iii) experienced discrimination (DISC-12), and iv) social functioning (Social Functioning Scale). Also, to investigate differences between the classes, socio-demographic, clinical, and person-related variables were examined.

**Results:**

Three classes were identified. The *Victimized and Perpetrating* class (34.4%) had the highest prevalence of discrimination, victimization, and perpetration, and intermediate scores on social functioning subscales. This class also experienced the most problems in other domains, such as psychosocial functioning and quality of life. The *Discriminated and Avoiding* class (36.4%) had moderate scores for discrimination, victimization and perpetration, and the lowest scores for social functioning and social support. The *General Difficulties* class (28.8%) had the lowest prevalence of discrimination, victimization, and perpetration, and the highest scores on social functioning.

**Discussion:**

These distinct classes offer new insights to mental health professionals in outpatient teams in in their aim to positively influence the patient’s social context during rehabilitation; this includes addressing the role of victimization, and indicates the relevance of distinctive approaches and the support needed for each class. Professionals may need to focus more on the impact of difficulties in their patients’ social context to adequately support them in the rehabilitation process.

## Introduction

People with severe mental illness are more likely to become a victim of crime than other citizens [1–4]. Studies in the Netherlands have reported prevalence rates for all types of crime victimization, ranging from 41.6–47% for outpatients in the previous year [2–5], whereas for violent crimes (e.g. physical or sexual assault) prevalence rates range from 17.1–22.5% [2–5]. When persons with severe mental illness experience criminal victimization, the number of incidents is often higher than for other citizens, i.e. they are more often a poly-victim [2]. Furthermore, most crimes tend to be committed in the individual’s own home. Correspondingly, the majority of perpetrators are familiar to the victim, e.g. it is often the (ex-)partner, neighbor, or roommate in the housing facility or inpatient setting [6, 7]. Criminal victimization can have long-term consequences and may harm a person’s trust in others, impair social relationships, and negatively affect their quality of life [4]; moreover, the accumulation of criminal victimization (poly-victimization) often indicates the accumulation of problems on other life domains [8].

In addition, individuals with severe mental illness are more likely to be perpetrators of a crime than members of the general population [1, 9]. However, for some individuals with severe mental illness, the roles of victim and perpetrator are interwoven, often making this relationship more complex than generally realized. A few studies have examined the association between victimization and perpetration in the same sample of persons with severe mental illness [9–11]. Childhood victimization may lead to perpetration later in life, and there is a strong overlap between victims and perpetrators. An individual may even be a victim and perpetrator in the same incident, making it difficult to unravel what has contributed to becoming a victim or a perpetrator [12].

Besides criminal victimization, people with severe mental illness also experience a considerable amount of discrimination and stigmatization. Brohan et al. [13] found that almost 70% of their sample perceived discrimination. Similar to victimization, the experience of discrimination can seriously affect an individual in their daily activities [13, 14], both leading to a lower quality of life, lower self-esteem, avoidance of social interaction (i.e. the ‘why try’ effect), unemployment, and an increase in symptoms [13–16].

It remains unclear how frequently discrimination and stigmatization occur together with victimization and perpetration in persons with severe mental illness and how these individuals participate socially. In particular, little is known about how victimization, perpetration, discrimination, and stigmatization interact in different groups of individuals with severe mental illness, or how this is related to social participation within these groups. Identifying these different patterns of victimization in community living, and their relationship with social functioning, is important for mental health professionals when supporting patients in their rehabilitation trajectories.

Although several psychiatric rehabilitation methods have been implemented and have shown significant improvements in role functioning and life satisfaction [17–19], many individuals with severe mental illness still face unemployment, poverty, social isolation, criminal offending, and victimization [20, 21]. Moreover, despite the high rates of victimization among individuals with severe mental illness and the consequences of this in their daily lives, this is rarely a structural topic of conversation in community-based mental health teams [22, 23]. It is reported that trauma treatment in a wider range of patients is more effective than previously thought [24]. Less recognized is the effect of the difficulties and traumatic events that persons with severe mental illness encounter throughout their lives. It can be assumed that incidents of victimization incidents and discrimination form a serious threat for participation and personal recovery [25]. Thereafter, increased understanding, acknowledgment of the adverse experience, and the learning of coping skills will better prepare individuals with severe mental illness for possible risks in future situations [26, 27].

Therefore, this study aimed to identify conceptually cohesive profiles in outpatients with severe mental illness based on their experiences of victimization and perpetration, discrimination and stigmatization, and social functioning. Our hypothesis was that we would find variations in victimization, perpetration, experienced discrimination, and social functioning rates. Furthermore, we expected these groups to vary in terms of socio-demographic, psychiatric, and other variables, such as social support, self-efficacy, and quality of life.

## Materials and methods

### Participants

In the current mental health care system in the Netherlands, many people with severe mental illness receive outpatient care from flexible assertive community treatment (F-ACT) teams. The F-ACT model is a flexible mode of ambulatory care delivery which allows to switch from crisis management or assertive community treatment to multidisciplinary treatment and individual case management when necessary [28]. In the present study, eight F-ACT teams from two mental health organizations participated; three teams were located in the north-west of the Netherlands and five in the south. Four teams had an urban catchment area, two were based around small cities, and two were in rural areas. Inclusion criteria for this study were: having a severe mental illness, according to the Diagnostic and Statistical Manual of Mental Disorders-IV (DSM-IV), aged ≥ 18 years, and willing to participate. Exclusion criteria were: aged < 18 years, insufficient comprehension of the Dutch language, unable to complete the interview due to cognitive impairment, florid psychosis or psychiatric crisis (i.e. having a serious relapse), psycho-organic disorder, and prolonged admission to psychiatric hospital or prison. From the eight teams (caring for 1527 patients), 133 patients met the exclusion criteria and the remaining patients (n = 1394) were eligible to participate.

Finally, 408 outpatients met the inclusion criteria and agreed to participate; these individuals were interviewed between March and August 2016. The response rate of 27% is similar to that of a large national Dutch study on victimization conducted by Kamperman et al., i.e. 29% [2]. Of the recruited 408 patients, 395 were finally included in the analyses; the 13 excluded patients had missing data on (at least) one of the primary outcome variables.

### Procedure

This study is part of a cluster randomized controlled trial in which the effectiveness of a novel intervention for victimization and societal participation was assessed. The study protocol was approved by the Medical Ethical Committee of the Elisabeth Hospital in Tilburg (NL53845.028.15) for all participating sites. The study was registered in the Dutch Trial Register (NTR 5585).

All patients received a letter and brochure with information about the trial, including details on the themes and timeframe of the study. All participants could withdraw from participation at any time for any reason. After a two-week consideration period, patients were contacted to provide them with more information (if required) and to ask if they were still willing to participate. When the patient agreed to participate, written consent was requested before the start of the interview. If the patient declined participation, this had no consequences for the care they received.

Data were collected during face-to-face structured interviews in a location of the participant’s choice, e.g., the patient’s home or the F-ACT office. Regular checks were made with the patient’s mental health professional to confirm whether the home environment was a safe place for the interview to take place (for both the patient and interviewer). Each interview lasted on average 75 minutes, after which the patients received a small financial compensation. In addition, the main mental health professional for each participating patient filled out a brief questionnaire, including the information described below (see ‘Measures’).

### Measures

To determine the classes, four concepts were taken into consideration: i) experienced discrimination, ii) victimization, iii) perpetration, and iv) social functioning. These measurements were chosen according to their usage in (inter)national mental health research and their acceptable psychometric properties.

**Experienced discrimination** was assessed by the Discrimination and Stigmatization Scale (DISC-12) [29]. The scale ‘unfair treatment’, or experienced discrimination, contained 22 items (α = 0.82). All items were answered on a four-point scale ranging from ‘no difference’ (0) to ‘a lot’ (3). A ‘not applicable’ answer was available when the participant was not involved in the described situation. Scores on the 22 items were summed, and a mean score was used in the analyses (minimum = 0, maximum = 2). Inter-rater reliability ranged from 0.62–0.95. Overall reliability was also adequate (α = 0.78) [29].

**Anticipated stigmatization** and **overcoming stigmatization** were also measured with the DISC-12. Anticipated stigmatization contained four items, and overcoming stigma two items. For each scale, all scores were summed and a mean score was used in the analyses.

**Victimization** was measured using the Dutch Safety Monitor, developed by the Dutch Ministry of Security and Justice [30], which is similar to the International Crime Victimization Survey [31]. In this larger Dutch questionnaire, the victimization section contains 15 crime incidents: burglary, theft from car, car theft, theft of other motorized vehicles, bicycle theft, (attempted) robbery, theft (other than previously categorized), sexual intimidation or assault, threats (of violence), physical assault, vandalism, identity fraud, fraud with buying/selling items/services, hacking, and cyber bullying. Car and motor theft items were not included in the scores on victimization because only very few participants owned a vehicle. For each incident, the participant was asked whether this had happened in the last year, yes (1), or no (0). All scores were summed, and a sum score (minimum = 0, maximum = 7) was used in the analyses. Although the Safety Monitor is the largest safety survey used in the Netherlands, and is the most reliable measure available, psychometrics were not available as the questionnaire is updated yearly and used for annual monitoring. Poly-victimization was defined as experiencing four or more different types of incident in the last 12 months [32], and was calculated for patients that reported at least one victimization incident during the last 12 months.

**Perpetration** was also assessed with the Dutch Safety Monitor. Regarding incidents of victimization, participants were asked whether they had been a perpetrator in the previous year. A sum score (minimum = 0, maximum = 7) was used in the analyses.

**Social Functioning** was measured using the Social Functioning Scale (SFS) [33]. This tool measures social functioning in seven domains: social engagement/withdrawal (time spent alone, initiation of conversations, social avoidance), interpersonal behavior (number of friends, quality of communication), pro-social activities (engagement in a range of common social activities), recreation (engagement in a range of common hobbies or interests), independence-competence (ability to perform skills necessary for independent living), independence-performance (performance of skills necessary for independent living), and employment/occupation (engagement in employment or structured daily activities). The SFS has good internal consistency (0.80) [33]. Item scores on all seven domains were summed, and a sum score (minimum = 574.50, maximum = 891.50) was used in the analyses.

Additionally, we included the following measures to further describe the classes.

Information from the main mental health professional: **general psycho-social functioning** was measured with the Health of the Nation Outcome Scale (HoNOS) [34]. This scale contains 15 items on which the professional scored the patient’s functioning on a scale ranging from ‘no problems’ (0) to ‘a lot of problems’ (4); a sum score was included in the analyses. The intra class correlation coefficient was 0.92, and the Cronbach’s alpha for the overall scale is 0.78 [35]. The mental health professionals were also asked to report the patient’s registered **psychiatric diagnosis** according to the DSM-IV, which was the DSM version used during inclusion. Clusters of diagnoses included in the analyses were: schizophrenia, other psychotic disorders (i.e.: brief psychotic disorder, delusional disorder, psychotic disorder due to a general medical condition, schizoaffective disorder, schizophreniform disorder, shared psychotic disorder, and substance-induced psychotic disorder), mood disorder, anxiety disorder, developmental disorder, substance use disorder, other Axis 1 diagnoses (i.e. in this sample: cognitive disorder, dissociative disorder, eating disorder, intermittent explosive disorder, pedophilia, alcohol-induced persisting amnestic disorder, impulse-control disorder, and somatization disorder), and personality disorder. All professionals received training on this instrument to enhance interrater reliability, as recommended by Ventura et al. [36]. The mental health professional was also asked to report whether the patient was ***av*oiding or stagnating in societal participation** on a scale ranging from (0) ‘not at all’ to (3) ‘yes, definitely’.

Two other measures from the Dutch Safety Monitor were **general feeling of unsafety**, answered with ‘yes’ and ‘no’, and **the expectation of becoming a victim in the next 12 months**, which was answered on a five-point scale ranging from ‘a really big chance’ to ‘a really small chance’.

**Social support** was derived from the Inventory of Social Reliance (ISR) [37]. This consists of 11 items on emotional and practical support scored on a four-point scale ranging from ‘almost never’ to ‘almost always’; a sum score was included in the analyses. The ISR is a frequently used questionnaire for individuals with severe mental illness and has good psychometric properties [37].

**Quality of life** was measured with the Manchester Short Assessment of Quality of Life (MANSA) [38]. The MANSA consists of 12 questions scored on a seven-point Likert scale ranging from ‘couldn’t be worse’ to ‘couldn’t be better’ and four questions that are answered with yes/no. Internal consistency is good (α = 0.81) [39]. Mean scores were included in the analyses.

**Self-efficacy** in mental health-related beliefs was measured with the Mental Health Confidence Scale (MHCS) [40], using a six-point Likert scale, ranging from ‘totally no confidence’ to ‘full confidence’. A sum score was included in the analyses. Cronbach’s alpha for the total scale is 0.91 [40].

**Sociodemographic characteristics**. The following socio-demographic variables were measured: age at the time of participation, gender (male ‘0’, female ‘1’), ethnicity (born in the Netherlands or not), living situation (living with family, on their own, supported living, other), marital status (married, not married, divorced, widow, cohabitation agreement), and employment status (benefits, retired, employed, other).

### Statistical analyses

Latent class analysis (LCA) was conducted to determine the underlying latent structure of the data. Although this is comparable to confirmatory factor analysis [41], in LCA the persons are grouped, rather than the items. We tested a series of latent class models (one to seven classes) to determine which model fitted the data best. Several indices were used to determine the model that best fitted the structure of the data and that were also theoretically and practically relevant.

First, the Bayesian information criterion (BIC) and the Akaike information criterion (AIC) with a penalty factor of three (AIC3) were used as goodness-of-fit indices [41, 42]. It was found that, in studies with few indicators and a moderate to large *N*, AIC often selects an unnecessarily complex model, making AIC3 a better alternative [43]. BIC was also included and was found to be a consistent information criterion in LCA [41, 43]. For both measures, lower values indicate a better fit of the model to the data.

Second, bivariate residuals were included to determining the number of classes to check for violation of the assumption of local dependencies between the included variables on which the clusters were based (i.e., discrimination, victimization, perpetration, and social functioning). If bivariate residuals are > 4, this implies that this assumption is violated [44].

Finally, class probabilities for the suggested solution were examined. The classification error was also considered, i.e. the chance that a patient was assigned to the wrong class. Thus, the ultimate class solution was based on the goodness-of-fit of the indices, classification errors, and bivariate residuals.

After identifying the number of classes, bias-adjusted three-step LCA was conducted to determine whether classes differed in sociodemographic, clinical, or other characteristics. This type of analysis consider the probability of belonging to all classes and, therefore, corrects the classification error [45]. Within this type of analysis, the ‘dependent option’ is an ANOVA-like test to examine differences across classes. Moreover, paired comparisons with a Wald-statistic were used to evaluate differences between pairs of groups. The LCA and other analyses were performed with Latent Gold 5.1 [41]. A significance level of 0.05 (two-tailed) was used for all tests.

## Results

### Sample characteristics

In the total sample (*N* = 395), the mean age was 45.4 (SD = 9.78) years, with 59% in the age range 30–50 years; 40.3% of the patients were female, 83.5% were born in the Netherlands, 80.2% lived independently, and 14% had paid employment. The majority of the participants were diagnosed with schizophrenia (26.8%), another psychotic disorder (24.6%), or personality disorder (14.7%). Analyses showed no significant difference between the respondents and non-respondents regarding age, gender, mental health care center, and F-ACT team.

### Latent class analysis

Firstly, model fit statistics were examined (Table 1). Although the AIC3 decreased with an increasing number of classes, the differences were small (± 10) after the fourth class, indicating that a more complex model offered no additional value. In the three-class solution, the BIC had the lowest value in the three-class solution.

**Table 1:** Fit indices for latent class analysis (*N* = 395).

No. of classes	Log-likelihood	BIC (LL)	AIC3 (LL)	Entropy R^2^	No. of parameters	Classification error
1	-3156.423	6414.487	6363.845	-	17	0.000
2	-3027.020	6197.533	6126.040	0.682	24	0.089
3 *	-2989.221	6163.788	6071.443	0.650	31	0.159
4	-2968.819	6164.837	6051.639	0.668	38	0.177
5	-2953.612	6176.274	6042.224	0.695	45	0.170
6	-2938.454	6187.810	6032.908	0.729	52	0.182
7	-2919.470	6191.695	6015.940	0.754	59	0.184

* Selected model

Secondly, in all calculated clusters, bivariate residuals for victimization and perpetration were above four. Although the most straightforward solution would have been to increase the number of clusters to an eight-cluster model or higher, this would have led to a small *n* per cluster, which was undesirable. Another solution was to allow for local dependencies between these two variables [41]. Correlation between perpetration and victimization was allowed, based on Choe et al. [1]. By allowing residuals to correlate, the three-class model provided the best solution (BIC = 6163.79, classification error = 0.159).

Finally, class probabilities for the three-class solution were high (averaging 0.79–0.91), indicating that individuals were assigned to the correct latent class. The three-class model was the most appropriate model, considering model fit statistics and theoretical implications.

### Description of the classes

**Fig 1:**
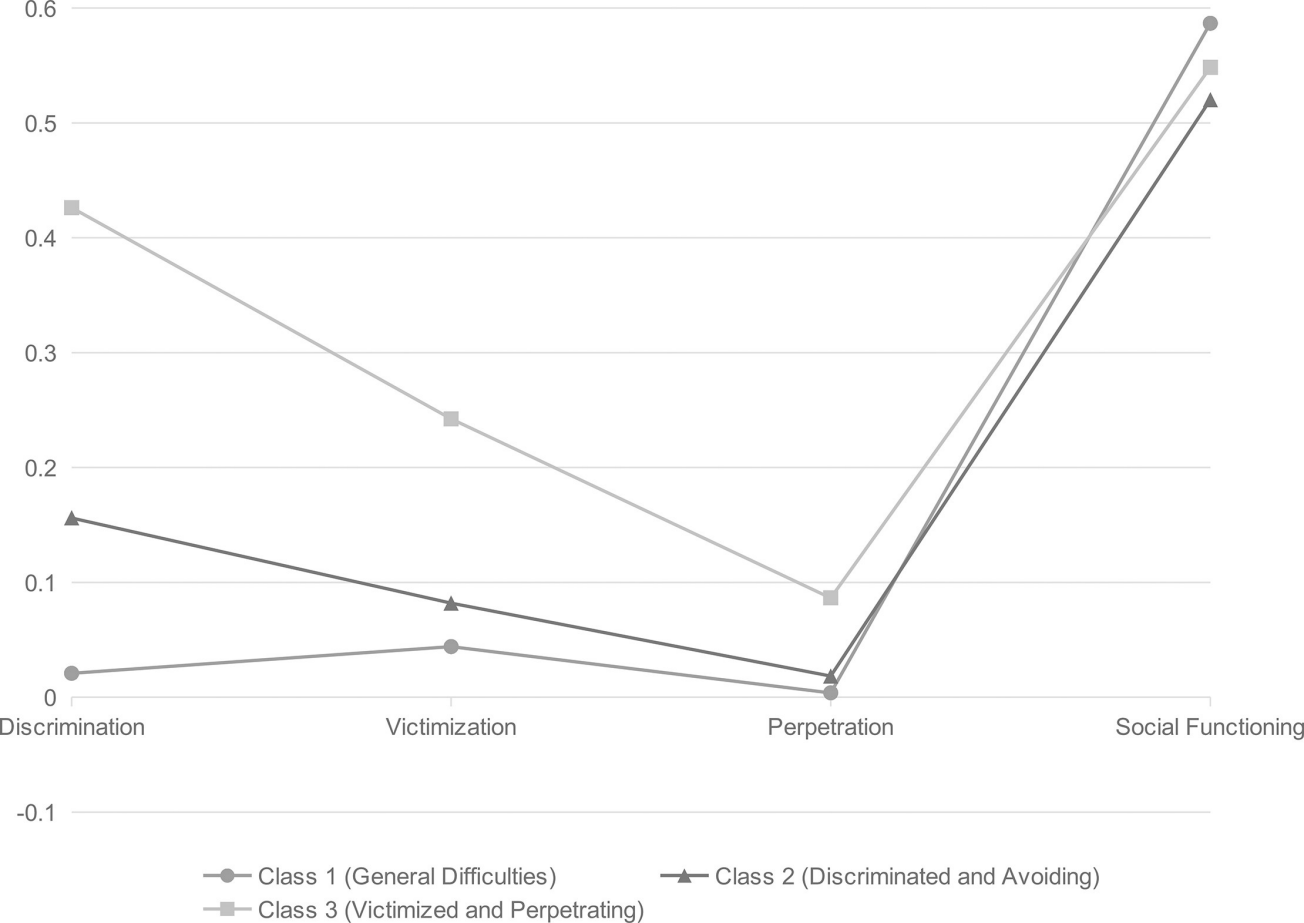
Profiles of the three classes based on discrimination, victimization, perpetration, and social functioning. (Standardized scores, *N* = 395).

A plot of the estimated probabilities of the three classes is presented in Fig 1 and the probabilities and scores of each item are given in Tables 2 and 3.

**Table 2:** Victimization and perpetration items per class.

	Full sample	Class 1: General Difficulties class	Class 2: Discriminated and Avoiding class	Class 3: Victimized and Perpetrating class			
	(*N* = 395)	(n = 114) (28.8%)	(n = 145) (36.8%)	(n = 136) (34.4%)	Wald	p-value	Group differences ^a^
Property victimization	19.2%	8.8%	9.4%	38.6%	29.428	0.000*	V>G,D
Personal victimization	21.5%	5.9%	7.1%	50.1%	46.897	0.000*	V>G,D
Digital victimization	15.7%	3.4%	5.9%	36.4%	34.074	0.000*	V>G,D
Poly-victimization ^b^	10.8%	2.3%	0.0%	20.5%	200573.313	0.000*	D<G<V
Total victimization	46.8%	24.8%	39.6%	73.0%	23.682	0.000*	V>G,D
Property perpetration	5.1%	0.0%	2.7%	12.1%	13601.182	0.000*	G<D,V
Personal perpetration	11.4%	0.9%	0.6%	31.7%	12.734	0.002*	G<V
Digital perpetration	10.1%	5.6%	6.4%	17.9%	8.594	0.014*	V>G,D
Total perpetration	17.5%	2.6%	11.3%	36.5%	8.799	0.012*	V>G
Anticipated stigmatization (DISC-12) ^c^	0.812	0.493	0.723	1.177	35.270	0.000*	V<G<D
Overcoming stigmatization (DISC-12) ^c^	1.186	1.192	0.898	1.488	15.055	0.001*	G<V, D>V

*Note*: p-value of Wald statistic

* p < 0.05

^a^ G: General Difficulties class, D: Discriminated and Avoiding class, V: Victimized and Perpetrating class, ns: no significant paired comparisons

^b^ Poly-victimization is presented as ‘victimization of which poly-victimization’ (n = 185

^c^ Mean scores are presented (minimum = 0, maximum = 3)

**Table 3:** Social functioning items per class.

	Full sample	Class 1: General Difficulties class	Class 2: Discriminated and Avoiding class	Class 3: Victimized and Perpetrating class			
	(*N* = 395)	(n = 114) (28.8%)	(n = 145) (36.8%)	(n = 136) (34.4%)	Wald	p-value	Group differences ^a^
Engagement	92.11	93.84	91.08	91.76	5.925	0.052	D<G
Interpersonal	118.71	124.75	115.04	117.57	13.243	0.001*	G>D,V
Independence—performance	109.74	111.40	107.81	110.40	5.658	0.059	D<G
Recreation	114.47	114.85	110.81	118.08	7.686	0.021*	D<V
Pro-social	105.14	108.00	101.09	107.08	10.800	0.005*	D<G,V
Independence—competence	107.28	109.28	106.36	106.58	5.541	0.063	G>V
Occupational	102.62	104.11	102.35	101.65	1.269	0.530	ns

*Note*: p-value of Wald statistic

* p < 0.05

For each subscale sum scores are presented, with 100 being set as an average for patients with SMI.

^a^ G: General Difficulties class, D: Discriminated and Avoiding class, V: Victimized and Perpetrating class, ns: no significant paired comparisons.

This first class (*n* = 114, 28.8%) experienced the lowest number of victimization incidents: 25% of this class had experienced one or more incidents in the past year (vs. 18% of the general population in 2015) and were (almost) never a perpetrator of an incident. Furthermore, 5.9% of the individuals in this class had experienced a personal victimization incident, compared to 2.2% of the remainder of the population [30]. For property victimization, these data are 8.8% and 12.2%, respectively. Therefore, this class is labelled the *General Difficulties* class. This class had the highest scores on the social functioning subscales ‘interaction’ and ‘pro-social’. In terms of anticipated discrimination, members of this class had significantly lower scores than the other classes.

The second class (*n* = 145, 36.8%) had a higher prevalence rate of experienced discrimination than the first class. In particular, this class had the lowest scores on overcoming stigmatization; this implies that individuals in this group felt they had the least skills in coping with discrimination. This class was further characterized by the lowest scores on all three social functioning subscales (‘interaction’, ‘recreation’, and ‘pro-social’), which differed significantly between the classes. This class is labelled the *Discriminated and Avoiding* class.

The third class (*n* = 136, 34.4%) had the highest prevalence of victimization and perpetration, and also had the highest scores for experienced discrimination and anticipated stigmatization. On the other hand, this class had the highest scores for overcoming stigmatization and on the ‘recreation’ subscale of social functioning (which contained items on the number of times the patient had read, repaired things, shopped, played an instrument, etc.). The average number of victimization incidents per year was 1.7 for this group (S1 Table), and > 50% of this class had experienced one or more personal victimization incidents in the previous year (including threats of violence, violence, and sexual intimidation/assault) as compared to < 10% in the other two classes. This class is labelled the *Victimized and Perpetrating* class.

Table 2 also gives the prevalence rate of poly-victimization in the three classes. For the *Victimized and Perpetrating* class, this means that of the group that reported one or more victimization incidents, 20.5% can be defined as a poly-victim. This accounts for 17.5% of the total *Victimized and Perpetrating* class (S2 Table).

### Differences in socio-demographic, clinical, and person-related variables

Of the sociodemographic variables, significant differences were found in age and living situation (Table 4). The *Victimized and Perpetrating* class contained the youngest persons (mean age 41.9 years), followed by the *General Difficulties* class (mean age 46.9 years), and the *Discriminated and Avoiding* class (mean age 47.5 years). Regarding the living situation, although the differences were small, the *Discriminated and Avoiding* class contained the most individuals that lived independently.

**Table 4:** Characteristics of patients with regard to the full sample and the three classes.

		Full sample	Class 1: General Difficulties -class	Class 2: Discriminated and Avoiding class	Class 3: Victimized and Perpetrating class			
		(*N* = 395)	(n = 114) (28.8%)	(n = 145) (36.8%)	(n = 136) (34.4%)	Wald	p-value	Group differences ^a^
Age (mean) in years		45.4	46.9	47.5	41.9	18.404	0.000*	V<G,D
Female		40.3%	38.3%	40.6%	41.6%	0.229	0.890	ns
Born in the Netherlands		83.5%	83.7%	84.0%	83.0%	0.036	0.980	ns
Living situation	Living with parents or family	4.3%	6.2%	2.2%	5.1%	2501.169	0.000*	
	Living on their own	80.2%	80.2%	82.1%	78.0%			
	Supported (independent) living	14.5%	13.6%	15.0%	14.6%			
	Other	1.0%	0.0%	0.8%	2.3%			
Marital status	Not married	66.5%	74.0%	62.8%	64.3%	9.505	0.300	ns
	Divorced	16.2%	13.3%	12.6%	22.7%			
	Married	14.0%	10.1%	19.3%	11.4%			
	Widow/widower	2.0%	0.9%	3.9%	1.0%			
	Cohabitation agreement	1.3%	1.8%	1.5%	0.7%			
Employment status	Benefits	72.4%	69.8%	68.0%	79.3%	4.331	0.630	ns
	Retired	1.3%	0.1%	2.9%	0.5%			
	Employed	14.0%	16.4%	16.4%	9.6%			
	Other	12.2%	13.7%	12.7%	10.6%			
Primary diagnosis	Schizophrenia	26.8%	37.2%	30.8%	13.8%	1136.088	0.000*	
	Other psychotic disorder ^b^	24.6%	39.3%	16.9%	20.3%			
	Mood disorder	12.4%	5.5%	21.6%	8.2%			
	Anxiety disorder	7.1%	3.0%	3.5%	14.3%			
	Developmental disorder	9.9%	2.6%	13.6%	12.0%			
	SUD	1.5%	2.4%	0.0%	3.0%			
	Other Axis 1 diagnosis ^c^	3.0%	3.3%	2.4%	3.5%			
	Personality disorder	14.7%	6.8%	11.3%	24.9%			
SUD		38.2%	34.3%	37.0%	42.9%	1.595	0.450	ns
Feeling unsafe (Safety Monitor)		52.6%	36.1%	54.5%	64.4%	14.649	0.001*	G<D,V
Expecting to be a victim in next 12 months (Safety Monitor) ^d^		3.00	2.24	2.97	3.66	10.529	0.005*	G<V
Avoiding social participation ^e^		1.27	1.24	1.14	1.43	3.006	0.220	ns
Stagnation in social participation ^e^		1.35	1.10	1.19	1.73	18.165	0.000*	V>G,D
Social support (ISR)^f^		27.59	29.15	26.00	27.99	7.110	0.029*	G>D
Self-efficacy (MHCS) ^g^		66.06	72.45	65.83	60.94	41.590	0.000*	G>D>V

*Note*: p-value of Wald statistic

* p < 0.05

SUD = substance use disorder

^a^ General Difficulties class, D: Discriminated and Avoiding class, V: Victimized and Perpetrating class, ns: no significant paired comparisons

^b^ Other psychotic disorders are: brief psychotic disorder, delusional disorder, psychotic disorder due to a general medical condition, schizoaffective disorder, schizophreniform disorder, shared psychotic disorder, and substance-induced psychotic disorder

^c^ Other Axis 1 diagnoses are: cognitive disorder, dissociative disorder, eating disorder, intermittent explosive disorder, pedophilia, alcohol-induced persisting amnestic disorder, impulse-control disorder, and somatization disorder

^d^ Sum scores are presented (minimum = 0, maximum = 12)

^e^ Item score is presented (minimum = 0, maximum = 3)

^f^ Sum scores are presented (minimum = 12, maximum = 44)

^g^ Sum scores are presented (minimum = 24, maximum = 96)

Diagnosis (Table 4) and psychosocial functioning (HoNOS) (Table 5), both filled out by the patient’s mental health professional, showed a significant difference between the three classes. Individuals in the *General Difficulties* class were significantly more likely to have schizophrenia or another psychotic disorder as a primary diagnosis compared with the other two classes. Although in the *Discriminated and Avoiding* class the highest percentage also suffered from schizophrenia (30.8%), individuals in this class were more likely to suffer from mood disorders (21.6%) and developmental disorder (13.6%) than those in the other two classes. In the *Victimized and Perpetrating* class, schizophrenia as a primary diagnosis was the least common of all the classes (13.8%). Most individuals in this class had a personality disorder, psychotic disorder (other than schizophrenia), or an anxiety disorder (including post-traumatic stress disorder) as a primary diagnosis.

**Table 5:** Psychosocial functioning (HoNOS) and quality of life (MANSA).

		Full sample	Class 1: General Difficulties -class	Class 2: Discriminated and Avoiding class	Class 3: Victimized and Perpetrating class			
		(*N* = 395)	(n = 114) (28.8%)	(n = 145) (36.8%)	(n = 136) (34.4%)	Wald	p-value	Group differences ^a^
Psychosocial functioning	Aggression ^b^	0.670	0.352	0.584	1.032	20.611	0.000*	V>G,D
	Self-harm	0.154	0.001	0.176	0.260	0.780	0.680	ns
	Substance use	0.791	0.462	0.770	1.098	12.188	0.002*	G<D,V
	Cognitive dysfunction	0.921	0.833	0.876	1.044	2.346	0.310	ns
	Physical disability	1.085	1.122	0.957	1.194	1.426	0.490	ns
	Hallucinations and delusions	0.783	0.908	0.710	0.756	1.604	0.450	ns
	Depression	1.124	0.765	1.140	1.409	17.765	0.000*	G<D,V
	Other symptoms	1.801	1.721	1.696	1.985	2.916	0.230	ns
	Personal relationships	1.490	1.214	1.308	1.922	20.540	0.000*	V>G,D
	Overall functioning	0.853	0.760	0.876	0.906	1.109	0.570	ns
	Residential problems	0.540	0.365	0.351	0.891	18.737	0.000*	V>G,D
	Occupational/recreational problems	0.737	0.501	0.654	1.024	12.035	0.002*	V>G,D
	Total psychosocial functioning ^c^	10.812	8.895	9.963	13.335	29.185	0.000*	V>G,D
Quality of life	Life as a whole ^b^	4.391	4.785	4.488	3.960	14.920	0.001*	V<G,D
	Job (or sheltered employment)	5.060	5.334	5.063	4.772	1.962	0.370	ns
	Unemployed/retired	4.126	4.233	4.619	3.610	5.609	0.061	G>D
	Financial situation	4.048	4.556	4.238	3.422	18.641	0.000*	V<G,D
	Number and quality of friendships	4.739	5.077	4.560	4.644	6.182	0.045*	G>D,V
	Leisure activities	4.545	5.028	4.671	4.003	20.012	0.000*	V<G,D
	Accommodation	4.939	5.566	5.043	4.303	25.528	0.000*	G>D>V
	Personal safety	5.154	5.437	5.391	4.664	20.944	0.000*	V<G,D
	People that you live with	5.283	5.581	5.487	4.831	6.498	0.039*	V<G,D
	Living alone	4.689	4.854	5.018	4.184	8.076	0.018*	V<G,D
	Sex life	3.942	4.177	3.891	3.811	2.261	0.320	ns
	Relationship with your family	4.763	5.521	4.677	4.217	22.491	0.000*	G>D,V
	Physical health	4.127	4.556	4.131	3.763	9.827	0.007*	D>V
	Mental health	3.932	4.441	3.885	3.555	14.665	0.001*	G>D,V
	Total quality of life ^d^	4.501	4.927	4.572	4.069	47.556	0.000*	G>D>V

Note: p-value of Wald statistic

* p < 0.05

^a^ G: General Difficulties class, D: Discriminated and Avoiding class, V: Victimized and Perpetrating class, ns: no significant paired comparisons

^b^ mean item scores are presented for each item of the HoNOS and MANSA

^c^ Sum scores are presented (minimum = 0, maximum = 31)

^d^ Mean scores are presented (minimum = 1.75, maximum = 6.55)

With regard to the overall score on the HoNOS, the *Victimized and Perpetrating* class had the highest score, indicating that they experienced the most problems in all life areas. Analysis of the specific items of the HoNOS (aggression, substance use, depression, personal relationships, residential problems, and motivation for treatment problems) showed a significant difference between the three classes. Again, on all these items, the *Victimized and Perpetrating* class had the most problems. The *Victimized and Perpetrating* class also scored the highest on stagnating on societal participation (assessed by the mental health professionals, implying that they experienced the most difficulties in participating socially and/or being socially active.

Regarding experienced social support, the *Discriminated and Avoiding* class scored significantly lower than the *General Difficulties* class, implying that individuals in this class experienced less social support (both emotional and practical). This was in line with the low scores on social functioning for this class.

In terms of the overall score on quality of life (MANSA), the *Victimized and Perpetrating* class scored the lowest, followed by the *Discriminated and Avoiding* class and *General Difficulties* class. Significant differences also emerged in several specific domains of the MANSA. The *Victimized and Perpetrating* class had the lowest scores for all items, except for the following items: ‘Having seen a friend in the last week’, and ‘Satisfaction with the number and quality of friendships’. On these latter items, the *Discriminated and Avoiding* class scored the lowest.

The three classes differed significantly in terms of self-efficacy and empowerment. Persons in the *Victimized and Perpetrating* class had the lowest scores for self-efficacy, followed by the *Discriminated and Avoiding* class and the *General Difficulties* class.

Finally, individuals in the *Victimized and Perpetrating* class scored significantly higher on expecting to become a victim compared with the *General Difficulties* class. With regard to feelings of unsafety, the three classes differed significantly; the *Victimized and Perpetrating* class had the most persons that felt unsafe (64.4%), followed by the *Discriminated and Avoiding* class (54.5%) and the *General Difficulties* class (36.1%).

## Discussion

### Principal findings

This study supports the existence of three distinct and meaningful patient profiles in relation to victimization, perpetration, discrimination, and social functioning, and provides information to help identify which patients might best benefit from what type of care. The group with the highest prevalence of victimization was the *Victimized and Perpetrating* class (34.4%). This class contained the lowest percentage of individuals with schizophrenia and had a relatively high percentage of individuals with a personality disorder. Furthermore, this class is characterized by problems in multiple domains, such as psychosocial functioning, self-efficacy, and quality of life. The class with moderate scores for experienced discrimination, victimization, and perpetration, the *Discriminated and Avoiding* class (36.4%), had the lowest scores on the subscales of social functioning (‘interaction’, ‘recreation’, ‘pro-social’). More specifically, individuals in this class undertook the least pro-social activities and experienced the least social support from their environment. Moreover, this group included more individuals with depression, bipolar disorder, and developmental disorder. The *General Difficulties* class (28.8%) had the lowest scores for experienced discrimination, victimization, and perpetration, and was comparable with the general population with regard to victimization and feelings of unsafety [30]. In this class, more individuals had schizophrenia or other psychotic disorders than in the other two classes.

### Strengths and limitations

A major strength of this study is that, to our knowledge, it is the first to examine whether classes are distinguishable in outpatients with severe mental illness with regard to experienced discrimination, victimization, and perpetration, and social inclusion. There is evidence that these concepts interact [23], and that these interactions differ within the large target group of outpatients with severe mental illness [13, 14, 46]. However, studies that formulated classes of outpatients with SMI examined heterogeneity only in relation to victimization and perpetration, or social recovery, but did not perform an LCA on all indicators [10]. Other studies that did perform LCA on victimization, included only adolescents [47]. Another strength of the present study is the detailed set of variables used to describe the classes, together with the relatively large sample size.

The following limitations should also be considered. First, there is a possibility of selection bias. For example, patients were excluded when they had insufficient understanding of the Dutch language, prolonged clinical admission (i.e. longer than the inclusion period), and florid psychosis or psychiatric crisis. Although we invited all patients in the participating teams and has a relatively long inclusion period to ensure representativeness, this might have led to a selection effect. We attempted to compensate for this with the 6-month inclusion period and by excluding as few patients as possible thereby keeping the sample as representative as possible. Due to privacy issues we could not examine whether patients who declined participation differed on all patient characteristics, however, the in the non-response analyses no differences were found. Furthermore, our sample was comparable with some characteristics in other studies done in persons with SMI. Participants in our sample scored 10.8 on psychosocial functioning, compared to a norm score of 11.4 [35], and 51.4% of our sample was diagnosed with schizophrenia or other psychotic disorder, compared to 64% and 67% respectively, in a large Dutch sample [48].

A second limitation is that, in the present analyses, only the current status of participants with regard to discrimination, victimization, perpetration, and participation were taken into consideration. It is known that outpatients with severe mental illness often switch between relapses and more stable periods in which there is room to regain social roles. Moreover, the overlap between victimization and perpetration may change over time [10]. Consequently, individuals may have switched classes over time, which leads to possibly varying scores on discrimination, victimization, perpetration, and social functioning.

Finally, since patients received financial compensation for their investment of time, this may have influenced the study results. The advantages/disadvantages of financial compensation continue to be discussed; it remains a controversial topic due to ethical issues, especially with marginalized groups and, in this case, with potentially complex/emotional interview topics [49, 50]. Moreover, compensation might influence the accuracy of our study results, as some patients might participate only for the financial reward [49]. On the other hand, participants tend to agree with financial compensation, recognizing both their investment of time and the value of their participation [51].

### Interpretation of findings

In the present study, the overall victimization rate was 46.8%, i.e. much higher than the overall perpetration rate of 17.5%. This is in line with previous studies that included outpatients with severe mental illness [2, 5, 6]. Our findings support the idea that persons with severe mental illness are more often victims than perpetrators of any type of crime. However, our results also indicate that, for some individuals, victimization and perpetration are interwoven. The *Victimized and Perpetrating* class showed perpetration rates ranging from 12.1% for property perpetration to 31.7% for personal perpetration, and victimization rates ranging from 36.4% for digital victimization to 50.1% for personal perpetration, indicating that, in this class, both victimization and perpetration rates are high compared to those in other studies on individuals with severe mental illness [2]. Simmons et al. [52] used the ecological model to explain why the accumulation or co-occurrence of victimization is problematic. A person has several layers of social context surrounding him/her (e.g. the individual, partner, family, and neighborhood) and when fear or victimization occurs in one or more layers, negative effects of this adverse event on the individual level (e.g. anxiety or depression) also accumulate. This is in line with the low rates of self-efficacy, quality of life, and psychosocial functioning, found in the *Victimized and Perpetrating* class in our study.

For the *Discriminated and Avoiding* class and the *General Difficulties* class, the highest victimization rates were found in property victimization; this is similar to previous studies in persons with severe mental illness [2, 5, 6]. The *Victimized and Perpetrating* class had the highest overall rates for victimization and, more specifically, personal victimization (50.1%) (i.e., sexual harassment or assault, threats of violence, physical assault). These high rates of personal victimization were not found in previous studies, not even in more specific groups such as inpatients or patients with substance use disorder, in which the prevalence rates are expected to be higher [4]. Thus, it appears that the *Victimized and Perpetrating* class is a group of patients that are negatively characterized in two ways; individuals in this class have a higher chance of not only being a victim of a serious criminal incident, but also being a perpetrator and having significant problems in several life domains (as well as in self-confidence and quality of life).

The present study found a high percentage (17.5%) of poly-victimization in the *Victimized and Perpetrating* class. To date, co-occurrence (or poly-victimization) has mainly been investigated in adolescents [8]. For adults with severe mental illness, one Dutch study found a prevalence rate of 9.9% in severe mental illness outpatients and 2.2% in the general population [2]. Although this rate seems similar, Kamperman et al. [2] defined a poly-victim as an individual that has experienced four or more incidents, irrespective of the type of victimization, i.e. a less strict definition than that used in the present study. According to their definition, 45.8% of the *Victimized and Perpetrating class* would be a poly-victim, and < 5% in the *Discriminated and Avoiding* class and *General Difficulties class*. In the present study, the *Victimized and Perpetrating* class comprises patients are not only at high risk of being victimized but are also a victim in a large number of independent criminal incidents.

The *General Difficulties class*, with the lowest prevalence rates in victimization and perpetration, had the highest percentage of persons with schizophrenia (48.4%), compared to the other two classes (36% and 18%, respectively). This is in contrast to the belief that, in persons with severe mental illness, a diagnosis of schizophrenia is mostly related to problems in certain life domains and, moreover, being violent and unpredictable [53]. Similar to our results, Gray et al. [54] found that, in secured mental health services, persons with schizophrenia were the least likely to commit a crime and those with personality disorder the most likely. In a study in which both victimization and perpetration were included in the analysis, they also found a lower victimization and perpetration rate in persons with schizophrenia compared to other diagnoses [55, 56].

All these results suggest that, in persons with severe mental illness, other risk factors play a role in the development of criminal behavior. Some of these other risk factors are well established, e.g. homelessness or supported living, and substance use [4]. However, in the present study, we found only small differences in the living situation within the classes with high and low victimization. It should be noted that, in our sample, < 1% had no permanent housing. Registered substance use disorder showed no significant difference between the three classes. However, the HoNOS item ‘problems with substance use’ showed a significant difference between the *Victimized and Perpetrating* class and the *General Difficulties* class, the former having more problems in this area; this indicates that, although the diagnosis of substance use disorder did not differ across the classes, mental health professionals assign more problems to substance abuse in this class.

### Clinical implications

The elevated prevalence of victimization and perpetration among persons in the *Victimized and Perpetrating* class, and the experienced discrimination in the *Discriminated and Avoiding* class, which in together comprise > 70% of our sample, suggests that outpatients with severe mental illness need more targeted support to prevent (re-)victimization and perpetration while regaining valued social roles. Although improvement in social functioning is a central aim of outpatient teams, the results of this study suggest a specific need for additional support to address difficulties related to community living.

Also, although the *General Difficulties* class had the least victimization of the three classes and had relatively low scores on psychosocial functioning, this class still has problems finding paid employment (16.4% have a regular job). This class seems to hit a ‘glass ceiling’ when trying to achieve paid employment. Therefore, mental health professionals should consider using more supported employment interventions (e.g. individual placement and support) in this class [57].

The *Discriminated and Avoiding* class had the lowest scores for social functioning items and experienced the least social support. Moreover, they were the least satisfied with the number and quality of friendships. When professionals stimulate these patients to participate socially, the focus should be on reducing the experienced discrimination and stigmatization. One intervention shown to be effective in reducing self-stigma is Narrative Enhancement and Cognitive Therapy, which focus on restructuring negative self-beliefs and enhancing the ability to narrate their life story [58, 59].

According to the mental health professional, the *Victimized and Perpetrating* class had the most conflicting personal relationships, of the three classes. Moreover, mental health professional s saw the most stagnation in social participation. This implies that, although they are socially active (as evidenced by their social functioning scores), they experience several difficulties in the process. Although professionals recognize problems in the social domain, there is room for improvement in outpatient mental health care. In practice, the focus of these teams tends to be on crisis management and less on rehabilitation [60], partly because of a fear of an increase in symptoms when addressing victimization [22].

To conclude, exposure to victimization related to social participation is an important factor in the lives of people with severe mental illness and encompasses more than psychological trauma alone. Therefore, an extensive form of trauma-sensitive and difficulty-sensitive care should be incorporated in outpatient mental health care[61], allowing room for taking (calculated) risks, as these are necessary in the social recovery process [62]. When addressing adverse incidents, calculated risks need to be acknowledged as part of the recovery process [25]. Therefore, particularly for the *Victimized and Perpetrating* class, mental health professionals should focus on preventing (re-)victimization in rehabilitation trajectories by addressing these experienced difficulties and turning them into calculated risks.

### Future research

Latent growth analyses over an extended period of time are valuable; they allow to examine whether patients with severe mental illness switch classes over time, and whether scores on victimization and other indicators vary over time. This may provide additional tools to help mental health professionals to individualize care and upscale/downscale the focus on rehabilitation as required. Additionally, future studies on rehabilitation should include other measures related to social functioning, that are more in-depth measures on social activities and possible issues. On the SFS, we found low variability in our sample. Furthermore, scores for social functioning were almost as high as those found in the general population [63]. Although this is a validated and often used measure for social functioning, the SFS focuses more on (daily and social) activities and to a lesser extent on the social capital of the network. This study provides a first insight into the heterogeneity that exists in victimization and social functioning: for future research, it is recommended to include social network and support measures to gain more insight into the number and type of social relations that patients have.

### General conclusion

This study provides further evidence for the high victimization rates in persons with severe mental illness, and it reveals three distinct subgroups that differ greatly in terms of discrimination, social participation, victimization, and perpetration. The results offer new insights for mental health professionals of outpatient teams, and support the need for a more individualized approach in rehabilitation trajectories for patients with severe mental illness. The need to acknowledge and incorporate experiences of perceived stigma, discrimination, and victimization in the treatment and rehabilitation plan is advocated in order to increase the number of successful rehabilitation processes and reduce victimization rates.

## Supporting information

S1 Table Scores of the three classes on discrimination, victimization, perpetration, and social functioning. (DOCX)

S2 Table Scores of the three classes on poly-victimization, both definitions. (DOCX)
