# Supplementary material for: Profiling of victimization, perpetration, and participation: A latent class analysis among people with severe mental illness
Source: PLoS One. 2018 Nov 30;13(11):e0208457. doi: 10.1371/journal.pone.0208457 (PMC6268008; doi:10.1371/journal.pone.0208457)
Supplement: S1 Table — (DOCX) [file pone.0208457.s001.docx]

**S1 Table. Scores of the three classes on discrimination, victimization, perpetration, and social functioning**

|  |  | Full sample | Class 1 (General Difficulties class) | Class 2 (Discriminated and Avoiding class) | Class 3 (Victimized and Perpetrating class) |
| --- | --- | --- | --- | --- | --- |
|  |  | (n = 395) | n = 114 (28.8%) | n = 145 (36.8%) | n = 136 (34.4%) |
| Experienced discrimination (mean) | | 0.420 | 0.042 | 0.312 | 0.853 |
| Victimization incidents | 0 | 53.2% | 75.2% | 60.4% | 27.0% |
|  | 1 | 24.3% | 19.8% | 26.5% | 25.7% |
|  | 2 | 11.6% | 4.2% | 9.5% | 20.2% |
|  | 3 | 5.8% | 0.7% | 2.8% | 13.4% |
|  | 4 | 3.3% | 0.1% | 0.8% | 8.7% |
|  | 5 | 0.8% | 0.0% | 0.1% | 2.1% |
|  | 6 | 0.8% | 0.0% | 0.0% | 2.2% |
|  | 7 | 0.3% | 0.0% | 0.0% | 0.7% |
|  | Mean | 0.884 | 0.309 | 0.573 | 1.697 |
| Perpetration incidents | 0 | 82.5% | 97.4% | 88.7% | 63.5% |
|  | 1 | 11.9% | 2.5% | 9.8% | 22.0% |
|  | 2 | 4.1% | 0.1% | 1.4% | 10.3% |
|  | 3 | 1.0% | 0.0% | 0.1% | 2.8% |
|  | 6 | 0.3% | 0.0% | 0.0% | 0.7% |
|  | 7 | 0.3% | 0.0% | 0.0% | 0.7% |
|  | Mean | 0.263 | 0.027 | 0.129 | 0.605 |
| Social functioning (mean) | | 748.551 | 760.496 | 739.404 | 748.338 |
