# Supplementary material for: Profiling of victimization, perpetration, and participation: A latent class analysis among people with severe mental illness
Source: PLoS One. 2018 Nov 30;13(11):e0208457. doi: 10.1371/journal.pone.0208457 (PMC6268008; doi:10.1371/journal.pone.0208457)
Supplement: S2 Table — (DOCX) [file pone.0208457.s002.docx]

**S2 Table. Scores of the three classes on poly-victimization, both definitions**

|  | Full sample | Class 1 (General Difficulties class) | Class 2 (Discriminated and Avoiding class) | Class 3 (Victimized and Perpetrating class) |
| --- | --- | --- | --- | --- |
|  | (n = 395) | n = 114 (28.8%) | n = 145 (36.8%) | n = 136 (34.4%) |
| Poly-victimization (definition A) | 17.7% | 4.0% | 2.3% | 45.8% |
| Poly-victimization (definition B) | 5.1% | 0.5% | 0.0% | 17.5% |

Definition A: 4 or more incidents in one year

Definition B: 4 or more separate incidents in one year
